# Clinician educators’ conceptions of assessment in medical education

**DOI:** 10.1007/s10459-022-10197-5

**Published:** 2023-01-20

**Authors:** D. A. Sims, F. J. Cilliers

**Affiliations:** 1 University of the Western Cape, 14 Blanckenberg Street, Bellville, South Africa; 2 Faculty of Health Sciences, University of Cape Town, Cape Town, South Africa

**Keywords:** Assessment, Assessor, Clerkship, Clinician educator, Conceptions, Assessment for licensure, Phenomenography

## Abstract

In pursuing assessment excellence, clinician-educators who design and implement assessment are pivotal. The influence of their assessment practice in university-run licensure exams on student learning has direct implications for future patient care. While teaching practice has been shown to parallel conceptions of teaching, we know too little about conceptions of assessment in medical education to know if this is the case for assessment practice and conceptions of assessment. To explore clinician-educators’ conceptions of assessment, a phenomenographic study was undertaken. Phenomenography explores conceptions, the qualitatively different ways of understanding a phenomenon. Data analysis identifies a range of hierarchically inclusive categories of understanding, from simple to more complex, and the dimensions that distinguish each category or conception. Thirty-one clerkship convenors in three diverse Southern settings were interviewed in three cycles of iterative data collection and analysis. Four conceptions of assessment were identified: passive operator, awakening enquirer, active owner and scholarly assessor. Six dimensions were elucidated to describe and distinguish each conception: purpose of assessment; temporal perspective; role and responsibility; accountability; reflexivity and emotional valence. Additionally, three characteristics that appeared to track the progressive nature of the conceptions were identified: professional identity, assessment literacy and self-efficacy. These conceptions encompass and extend previously described conceptions across different educational levels, disciplines and contexts, suggesting applicability to other settings. There is some evidence of a relationship between conceptions and practice, suggesting, together with the hierarchical nature of these conceptions, that targeting conceptions during faculty development may be an effective approach to enhance assessment practice.

**Supplementary Information:**

The online version contains supplementary material available at 10.1007/s10459-022-10197-5.

## Introduction

Assessment drives learning, but, as Boud (1995, p.36) writes, “The fundamental question is, ‘What kind of learning?’” Evidence of the undesirable learning effects of assessment is abundant (see Cilliers et al. (2010); Cilliers et al., (2012a, 2012b); Cilliers et al., (2012a, 2012b) for a summary and evidence of the effects of summative assessment), that of desirable learning effects less so. Undesirable learning effects of assessment in medical education, particularly assessment in a system where graduation from medical school leads to supervised internship and subsequent licensure for independent practice as a general practitioner, have consequences not only for students but also patient care and public safety.

Adapting assessment practice to ensure more desirable learning effects is a longstanding goal in higher and medical education (Black & Wiliam, 1998; Gibbs & Simpson, 2004; Joughin, 2010; Swan Sein et al., 2020). Programmatic assessment (Schuwirth & van der Vleuten, 2019) is a systematic response to this challenge. Whether such an approach is adopted or, as in many settings, change is undertaken more piecemeal, a key player in the assessment game is the individual involved in designing assessment. Designing assessment is essentially a behaviour and improving assessment practice, a behaviour change challenge. The behaviour of individual assessors during the design of assessment will be influenced by numerous factors, not least their conceptions of assessment.

## Background

Conceptions, the object of study in phenomenography, are defined as, “Experiential descriptions, that is, content-orientated and interpretative descriptions of qualitatively different ways people perceive and understand their reality” (Marton, 1981, p. 177)—in this study, assessment. Conceptions have also been described as ‘ways of conceptualizing’, ‘ways of experiencing’, ‘ways of seeing’, ‘ways of apprehending’, ‘ways of understanding’ (Marton & Pong, 2005).

Conceptions of knowledge, of teaching and of learning are widely written about, conceptions of assessment less so. The nature of teaching practice has been shown to be related to conceptions of teaching (Kember, 1997; Trigwell et al., 1999). Support exists for such a link between conceptions of assessment and its practice (Box et al., 2015; DeLuca et al., 2019; Govaerts, et al., 2013; Watkins et al., 2005). Changed conceptions appear to contribute to effective changes to educational practice (Ho, 2000; Ho et al., 2001). Attending to conceptions would thus seem a likely candidate target for successful assessment practice change.

The known conceptions of assessment of educators in basic education (school), higher education (university) and medical education settings are summarised in Table 1.

**Table 1:** Conceptions of assessment in different educational settings

Basic education	Higher education	Medical education
- Assessment as “irrelevant” (little or harmful purpose) - Assessment for “school accountability” (quality control) - Assessment for “student accountability” (summative assessment) - Assessment for “improvement” of student learning and teaching (formative assessment) From Brown (2004)	- Assessment as “knowledge production” (teacher-centred) - Assessment as “knowledge construction/transformation” (learning-centred) From Samuelowicz and Bain (2002) - Assessment as “reproduction” (content-reproduction) - Assessment as “transformation” (student-development) From Postareff et al. (2012) - “Status quo” (teacher-centred; content-reproduction) - “Awareness” (content- and learning-centred) - “Development (shared autonomy; learning-centred) From Halinen et al. (2013)	- Psychometric: tailored vs. standardised assessment - Socio-constructivist: self- vs. externally-regulated learning - Agency (student-regulated learning) - Mutuality (student- and teacher-regulated learning) - Objectivity (externally-regulated assessment) - Adaptivity (externally- and clinically-regulated assessment) - Accountability (clinically-regulated assessment) From de Jonge et al. (2017)

These conceptions are those of educators working in Australia, Finland and The Netherlands. While not all of these papers explicitly used phenomenography or were about conceptions more broadly, many described one or more dimensions of a conception of assessment

Conceptions are typically represented by different categories of description and dimensions that describe and illustrate those categories (Svensson, 1997). Discernible dimensions of existing conceptions include an accountability or quality control purpose (Brown, 2004; de Jonge et al., 2017); and a teacher-centred and knowledge-reproduction or student-centred and learning-related purposes for assessment (Brown, 2004; de Jonge et al., 2017; Halinen et al., 2013; Postareff et al., 2012; Samuelowicz & Bain, 2002). The relationship between existing descriptions of conceptions of assessment has not, however, been clarified. Whether conceptions of educators hold across contextually different settings has also not been explored e.g., from higher education to health professions education or from settings in the global North to those in the global South. Finally, there is little to suggest how knowledge of these conceptions can be used in interventions to bring about changed assessment practice. To start exploring these issues, our main research question was: What are clinician educators’ conceptions of assessment in a range of Southern settings? A secondary question was: How do these conceptions relate to existing conceptions of assessment? To address these, we undertook a phenomenographic study of clinician educators’ conceptions of assessment in exit-level medical education.

## Research methods

### The context of the study

Medical education is inextricably context-bound, yet the significant epistemic and pragmatic differences between contexts are seldom addressed explicitly. “Southern” refers to settings within the “global South” in contrast to settings in the “global North”, descriptors that are conceptual rather than geographical (Connell, 2014). There is no simple way to define “Southern-ness”. In part, it has to do with highlighting the fallacy of hegemonic assumptions that what happens in “Northern” settings holds true worldwide. This ignores the cultural and linguistic diversity and socio-economic realities that characterise the global South, including uneven access to services like health and education; significant levels of poverty (Table 2); and the persistent debilitating impact of coloniality (Naidu, 2021b). South-North differences also manifest as global power hierarchies in knowledge production, which has theoretical and practical implications. A subset of countries in the North continue to dominate the HPE literature (Maggio et al., 2022) and current literature on conceptions of assessment reflects this, originating from Northern settings. This dominance may be due to the relationship between resourcing and clinical and educational workload (Table 2), but may also result from research that does not resonate with Northern perspectives being less likely to be valued and published (Gosselin et al., 2016). Ultimately, we need to be careful not to perpetuate colonially-constructed divides such as a North/South binary (Paton et al., 2020); rather, the point we are trying to make is that context is crucial and ecologically-valid perspectives are important, necessary and legitimate (Naidu, 2021a, 2021b).

**Table 2:** Comparing contexts in which medical education takes place for the sites of this study and countries in the global North with prolific outputs of HPE research^1^: Crude measures of healthcare system efficiency and disease burdens (WHO, 2020)

Indicators	USA	Canada	UK	Nether-lands	Aus-tralia	South Africa	Mexico
*Economic and educational indicators, where schooling takes place*							
PIRLS: international achievement in reading (2016)^2^	549	–	559	545	544	320	–
TIMMS: countries average mathematics and science achievement (2019) ^2^	528	518	523	528	524	364	–
GINI co-efficient (2022) ^3^	41.1	33.3	35.1	28.1	34.1	63.0	45.4
Per capita GDP (USD)	63.6	43.3	41.1	53.4	51.7	5.7	8.3
*Resourcing and staffing of healthcare, where medical education takes place*							
Per capita health expenditure (USD)	10 921.01	5048.31	4312.89	5335.30	5427.46	546.69	540.37
Density of medical doctors (per 10 000 population)	26.1	23.1	28.1	36.1	36.8	9.1	23.8
Density of nursing and midwifery personnel (per 10,000 population)	145.5	99.4	81.7	111.8	125.5	13.1	24.0
*Burden of disease, that clinician educators in medical education contend with*							
Maternal mortality ratio (per 100 000 live births)	19	10	7	5	6	119	33
Under-five mortality ratio (per 1000 live births)	7	5	4	4	4	34	13
Neonatal mortality rate (per 1000 live births)	4	3	3	2	2	11	8
New HIV infections (per 1000 uninfected population)	0	0	0	0	0.04	4.94	0.08
Tuberculosis incidence (per 100,000 population)	3	5.6	8	5.3	6.6	520	23
Life expectancy at birth (both sexes; years)	78.6	82.8	81.4	81.6	82.9	63.6	76.6

^1^To illustrate the health professional education (HPE) North–South gap; the vast majority of HPE outputs were produced by “Northern” countries, with the USA, UK, Canada, Australia and Netherlands accounting for the top 5 country affiliations for medical education publications (Doja et al., 2014; Maggio et al., 2022; Rotgans, 2011; Tutarel, 2002)

^2^Trends in International Mathematics and Science Study (TIMMS) and Progress in International Reading Literacy Study (PIRLS) are international assessments, currently taken in 70 countries since 1995, that monitor trends in student achievement in mathematics, science and reading, and are used as an indicator of the quality of basic education systems; with lower scores representing poorer academic performance

^3^The Gini co-efficient, index or ratio, a measure of statistical dispersion, measures income or wealth inequality; with lower values as more equal and higher values as less equal (e.g. a Gini co-efficient of 60.0 means that the richest 20% of a population holds 80% of all income)

This study, then, was conducted at three medical schools in two Southern settings, South Africa and Mexico, selected to represent intra- and inter-country diversity, for instance, colonial histories (Dutch, British, Spanish) and so languages of instruction at the universities (those of the coloniser, not the colonised). The structuring of higher education in these countries reflects neo-colonial models and power hierarchies that, for example, see the uncritical adoption of innovations like competency frameworks crafted in the North; or universities striving to have graduates recognised by countries in the United Kingdom, Europe and the USA. South Africa is still recovering from the legacy of Apartheid, with persistent fractures resulting in recent student activism and violent upheaval in universities. Socio-economic, educational and health indicators, which impact on where students come from, where faculty teach and where healthcare is provided, also differ greatly between the research settings and those settings that contribute most prolifically to the HPE literature (Table 2).

For differences at an institutional-level, clinician educators were sampled from urban campuses of three universities: two public, research-intensive, but socio-culturally different, universities in South Africa (university 1 and 2) and one private university in Mexico (university 3). The first South African university was historically Afrikaans-language and Apartheid-aligned; the second English-language and liberal; and the third Mexican university was Spanish-language. At both South African medical schools biopsychosocial and Primary Health Care educational models are followed, and at the Mexican medical school, a patient-centred approach. Medical programs at all three universities admit students directly from high school and are six years in duration. In South Africa, students graduate after six years and then undertake a further two-year internship and a third year of community (social) service. In Mexico, the final preservice year consists of internship and social service for medical students. In both contexts residency training is optional after graduation and social service. In these settings, final year assessments evaluate both theory and clinical competency, leading to a variety of assessment methods used (e.g., written examinations, portfolios, OSCEs).

In these programs, clinician educators—who we will call “clerkship conveners”—are medical specialists responsible for overseeing educational and assessment practice in clinical placements. In these Southern settings, assessment in a clinical clerkship is typically overseen by an individual, as opposed to a larger assessment committee. These individuals may be (solely) responsible for the conceptualisation, design, implementation and decision-making (e.g., pass/fail decisions) of assessment practices. The clerkship convener is therefore a key role player in assessment. This makes the individual and their conceptions, important units of intervention in any attempts to enhance assessment practice.

All clerkship conveners from final year clinical placements at the three universities were invited to participate in the study. A total of thirty-one (31) clerkship conveners from different specialities (family medicine, paediatrics, neonatology, obstetrics and gynaecology, internal medicine, surgery, public health) were interviewed based on their availability (31/80 = 38.75%). Interviews, each 45–60 minutes long, were conducted in the language of preference of participants. If necessary, translation by an expert in both the language and educational context took place before analysis. An interview guide was adapted throughout the study due to the iterative nature of data collection and analysis, leading to an evolving guide with basic and more specific prompts (see appendix). Ethical clearance was obtained from participating universities and informed consent from participants.

### Phenomenography

Developed in Sweden in the 1970-1980s to study student learning in Higher Education (Dahlgren, 1975; Marton, 1981; Svensson, 1977), phenomenography is used widely geographically and across research fields (Åkerlind, 2018; Pang & Ki, 2016; Rovio-Johansson & Ingerman, 2016; Tight, 2016), including medicine, health care and health professions education (See: Barnard et al., 1999; Sjostrom & Dahlgren, 2002; Stenfors‐Hayes, et al., 2013).

**Fig. 1:**
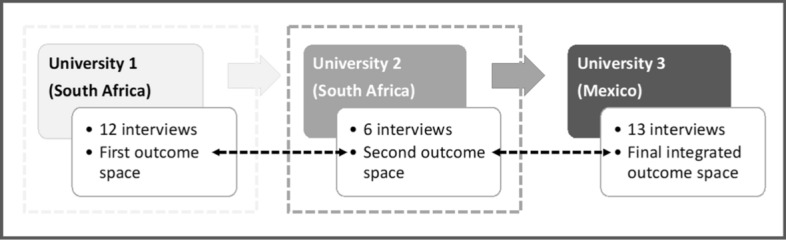
Sequential sampling and iterative data collection and analysis leading to outcome space development. Thirty-one semi-structured interviews of clerkship conveners responsible for final year assessment practices took place at three diverse universities in Southern settings. University 1: The first round of 12 interviews were analysed as a complete dataset, leading to the development of the first outcome space. University 2: The second round of 6 interviews were analysed together before comparing them to the previous data set, leading to a second outcome space that collated both datasets. University 3: The third round of 13 interviews were analysed as a dataset and subsequently compared to the previously collected datasets. This resulted in a final consolidated outcome space which encompassed all the data collected

In contrast to typical approaches to phenomenography, three rounds of data collection and analysis from three diverse Southern settings progressively developed our dataset and findings over time (see Fig. 1). Clerkship conveners’ conceptions of assessment were organised into an outcome space which is defined as, “The full range of possible ways of experiencing the phenomenon in question, at this particular point in time, for the population represented by the sample group collectively” (Akerlind, 2005, p. 323). An outcome space structures and describes conceptions as distinct, but interrelated descriptive categories, each a unit of meaning representing researchers’ abstractions from data which emerged during the interview and analysis processes (Svensson, 1997). These categories contain dimensions, or salient features, that further describe and illustrate different aspects of the conceptions (Marton & Pong, 2005).

While conceptions are treated as distinct for the purposes of description, they exist along a porous, hierarchical continuum. Categories are “hierarchically inclusive” (Stenfors-Hayes et al., 2013, p. 263) i.e., logically related to one another such that subsequent categories encompass previous categories, with the highest level representing the most sophisticated way of understanding a phenomenon (Stenfors-Hayes et al., 2013; Tight, 2016).

The seven-step analytical process described by Stenfors-Hayes et al. (2013) was adapted as data collection, and subsequent iterative analysis, took place in three rounds (see Fig. 1). Data from each round was first analysed inductively, treated as its own complete dataset, before deductively comparing it in rounds two and three to previous datasets and outcome spaces; allowing for sequential synthesis and refinement. The third and final integrated outcome space encompassed all the data collected.

While pragmatic convenience sampling was employed in each setting, theoretical sufficiency and conceptual depth were achieved through the rigorous and iterative data collection and analysis processes (Sims & Cilliers, in press). Theoretical sufficiency is demonstrated through the thick and rich descriptions of resultant conceptions, as supported by participant quotations (Braun & Clarke, 2021). In reflecting on the criteria for conceptual density, the multiple data-sets, multi-dimensional nature of the findings, strong resonance with existing theory—while adding variations and novelties—along with the high level of theorisation support our claim of sampling adequacy (Nelson, 2017). Our novel, progressive approach to phenomenography reflects our commitment to incorporating data from three distinctive Southern sites. As data was iteratively collected and analysed in multiple rounds for developing theoretical sufficiency and overall rigor, so the progressive evolution of the outcome space through three cycles took place.

Personal reflexivity was practiced through the keeping of a research journal and regular critical discussions between the authors, examining assumptions and beliefs, and how they may have influenced the research. Epistemological reflexivity was exercised through intentional sampling for diverse participants, interpretation remaining true to participants’ experiences and understandings (demonstrated through their own words), and use of an independent peer to review our findings through blind selection of random transcripts.

### The process of progressive phenomenographic analysis

The process of phenomenographic analysis is outlined below. Both authors independently undertook steps 1–4 with the initial dataset. The codes were compared and then author DS undertook steps 5–7 to craft the first outcome space, which went through several cycles of discussion and refinement, reviewing coding and associated data. The analysis of each stage informed data collection and analysis in subsequent stages. DS undertook analysis of the second and third datasets across several cycles of discussion and refinement with FC. Towards the end of the development of the final outcome space, a third reviewer (LP) reviewed one transcript from each cycle of interviews against the final outcome space. While the first outcome space resonated with literature on conceptions of teaching, as data collection and analysis progressed, new dimensions emerged as did unique categories of description.

Step 1, familiarisation, began with the transcription of the recorded interview and extended to repeated readings of the transcripts. This process enabled us to become intimately familiar with the data. Importantly, transcripts were hermeneutically read through two lenses; the first considered the intention and meaning of the *individual*, and, the second analysed the transcript apart from the individual in the *collective* “pools of meanings” (Åkerlind, 2005; Collier-Reed & Ingerman, 2013; Dortins, 2002; Marton, 1981, 1986, p. 43). While eventual categories of description need to accurately and authentically reflect participants and reality, they do not singularly represent an individual’s conceptions, but rather are collective profiles of conceptions (Dortins, 2002).

Step 2, compilation, entailed the selection and marking of interesting, significant and relevant words and phrases into codes (units of meaning), while Step 3, comparison, involved noting similarities and differences in the data, specifically the codes.

Step 4, grouping, involved grouping similar and separating differing codes, leading to the development of preliminary categories of description and their dimensions. These categories represented the varied ways of experiencing and understanding a phenomenon i.e., the range of conceptions, and were then structured into a tentative outcome space. This process drew on the relationships between categories and their dimensions so as to reflect the life world of the participants (Stenfors-Hayes et al., 2013). Analysing smaller subsets of data each round allowed for more efficient grouping and structuring of the emerging outcome space.

Step 5, articulation, re-examined, and rearticulated, if necessary, the codes and proposed categories so that each articulation represented the essential meaning of a code and category, while Step 6, labelling, entailed naming the categories and the dimensions that contributed to and illustrated the conception to reflect the essence of each (see Table 3).

**Table 3:** Defining dimensions, the salient features that distinguish categories of description from one another, and characteristics associated with, but may not be an integral feature of, conceptions of assessment

*Dimensions of conceptions of assessment*
*Purpose of assessment* Includes (1) overall notions of assessment as either a concrete (practical) or a abstract (a tool to guide student learning) task; (2) how participants perceive its goal (administrative, psychometric and/or moral and social); (3) how it is used (summatively and/or formatively); (4) its resultant learning effects or outcomes (obtaining a grade, reproducing knowledge, developing competencies, impact on curriculum and health services); as well as (5) its horizon as local (a mark for a single student in a single course) or more global (a tool for student learning over a program and beyond)
*Temporal perspective* Related to the purpose of assessment; speaks to the practice and impact of assessment; ranging from short- (a single course or a “today” focus) to long-term (a program, lifetime or field; a “tomorrow” focus)
*Role and responsibility* Describe how participants saw themselves as assessors and clerkship assessment conveners (sense of ownership and involvement: a merely mechanistic operation or a thoughtful, intentional initiation): institutional administrator, disciplinary content-expert, educator (facilitator or learning guide, role model), leader
*Accountability* Who or what does assessment practice impact on and whose imperatives should be prioritised: the institution, profession and discipline, student (and program/curriculum) and/or the patient and society?
*Reflexivity* Metacognitive thinking around assessment. Passive or uncritical acceptance of practices; questioning or critical reflection of own assessment thinking and practices; basing their assessment thinking on assessment principles and theory/scholarship
*Emotional valence* The emotions associated with how participants expressed their views of assessment and its outcomes, ranging from negative to positive
Associated characteristics
*Assessment literacy* The level of technical assessment knowledge and understanding e.g., principles for quality assessment practice that participants implied or explicitly articulated; ranging from limited to developing to advancing to sophisticated
*Professional identity* Refers to how participants represented themselves professionally; such as solely a clinician, clinician-educator (tensions or conflicted vs. balanced or equal), educator or scholarly assessor
*Self-efficacy* The degree of confidence and competence participants believe they possess for practising assessment (designing, implementing, decision-making, etc.); ranging from low to high

Step 7, contrasting, compared the similarities and differences between categories such that categories are described by what they *are* and what they are *not*. This assists in distinguishing each category from another. Importantly, with each separate round of data collection and analysis, data subsets were first treated as complete datasets in and of themselves, before being analysed with and against previous data subset/s and outcome space/s, resulting in the development of a distinct outcome space for each round. With each iteration, the outcome space became more complex and nuanced, as seen in more and richer categories of description and dimensions and speaking to the achievement of conceptual depth and theoretical sufficiency. The third outcome space represented the entire data set in a final, integrated outcome space.

## Results

**Fig. 2:**
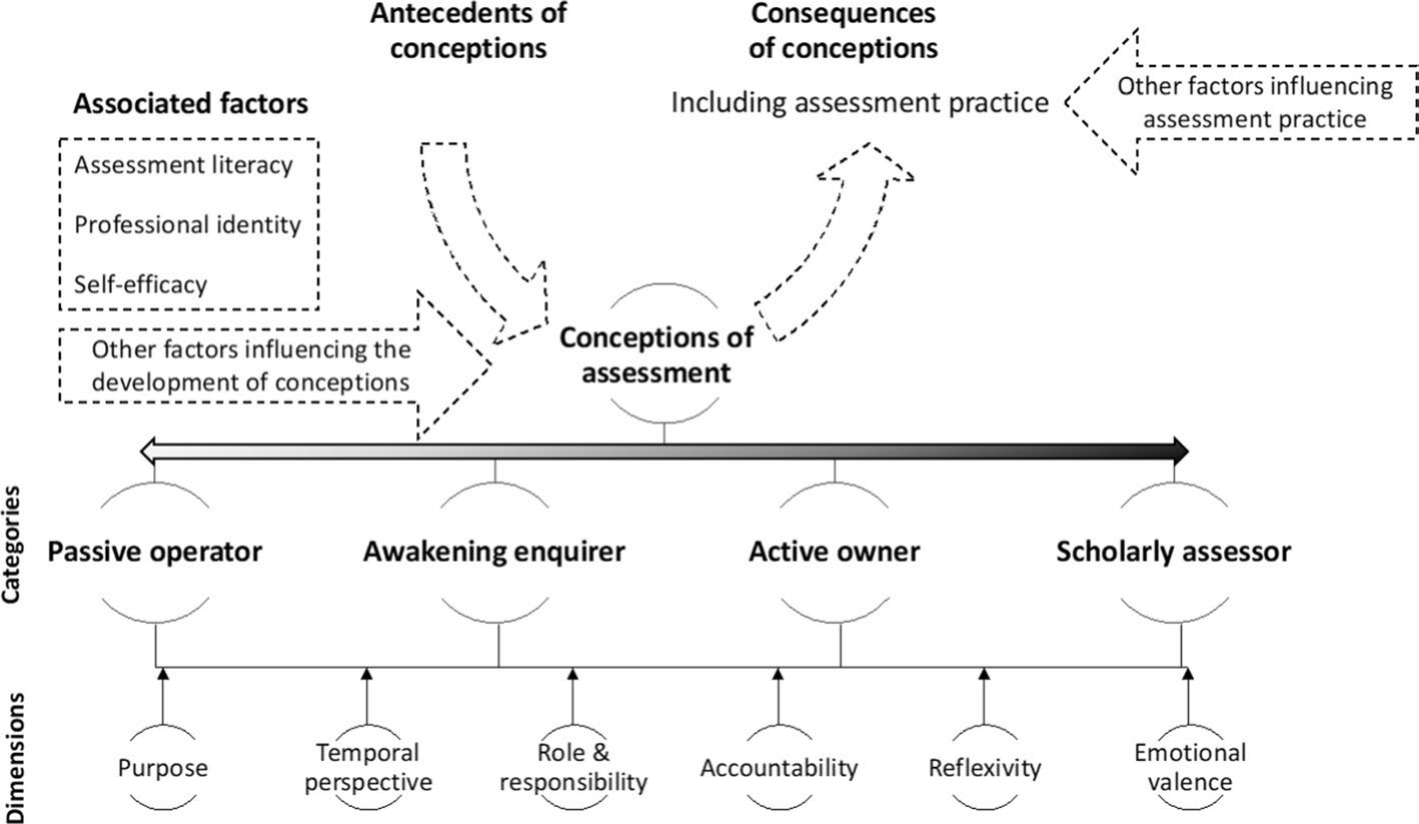
Conceptions of assessment. Clerkship conveners hold a range of conceptions of assessment characterised by four categories of description with six dimensions. Three associated factors appear to likely moderate, and be moderated by, said conceptions, yet the underlying mechanism of action of conception formation, development and enactment remain to be explored. Conceptions of assessment may manifest in related assessment practices, however additional factors also influence the practice of assessment

Analysis of data from university 1 yielded an outcome space consisting of three conceptions i.e., undirected; content focused-reproduction directed and competency and conceptually focused-application directed, with three dimensions for those categories i.e., purpose; learning effect and technical knowledge. This informed subsequent data collection at university 2, which in turn yielded an outcome space that had three conceptions i.e., detached practitioner; emerging equilibrium and engaged educator, with four dimensions i.e., purpose; temporal perspective; assessment literacy; identity and role. Data collection at university 3 allowed for further development and refinement. The final outcome space was organized into four conceptions of assessment with six dimensions, which incorporated all previous data (Fig. 2 and Table 4). As there were no discernible particularities by institution, notwithstanding the differences in context, data will not be reported by institution.

**Table 4:**
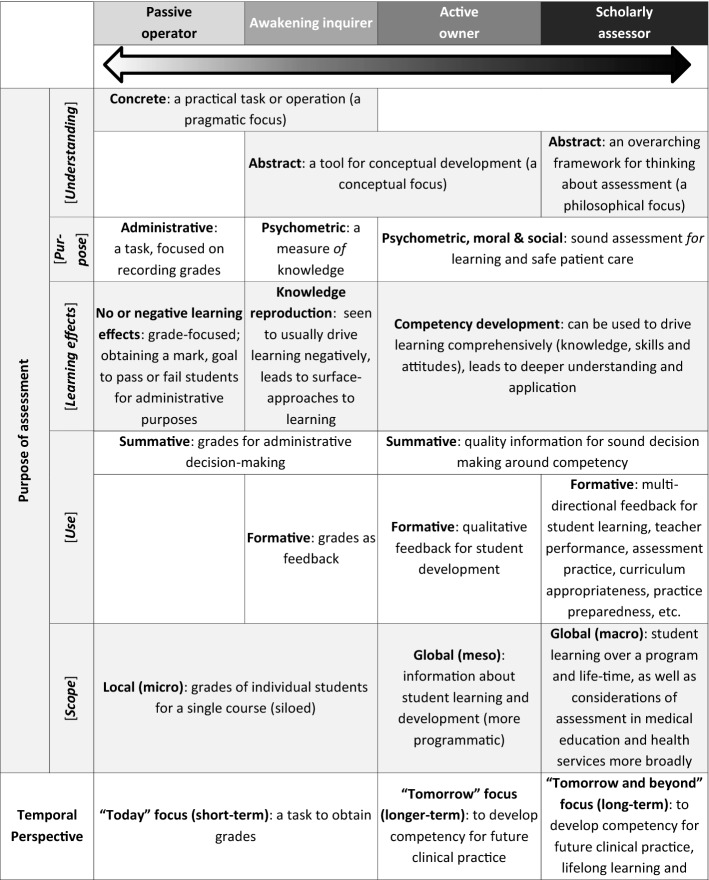

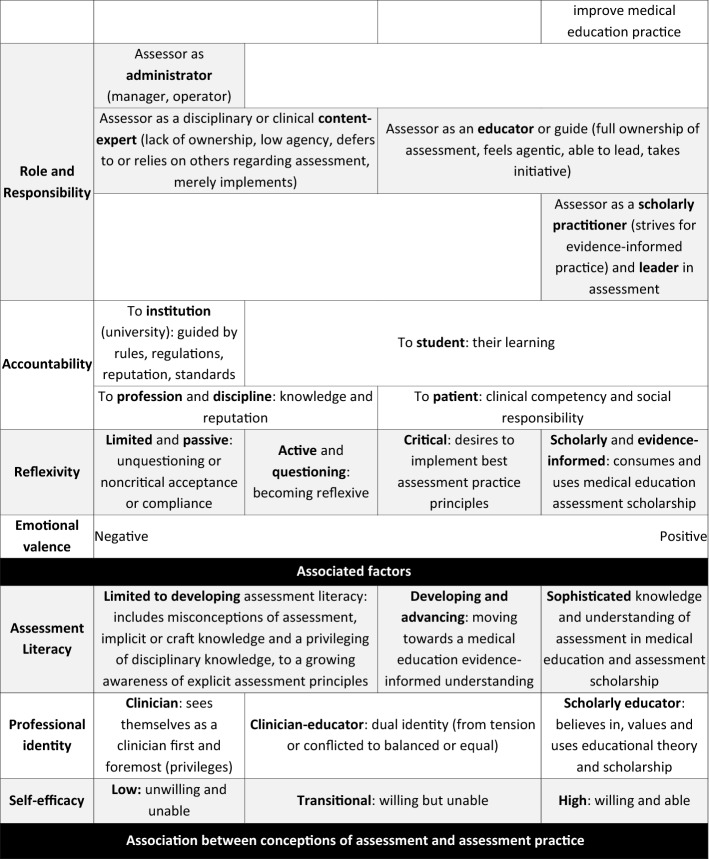

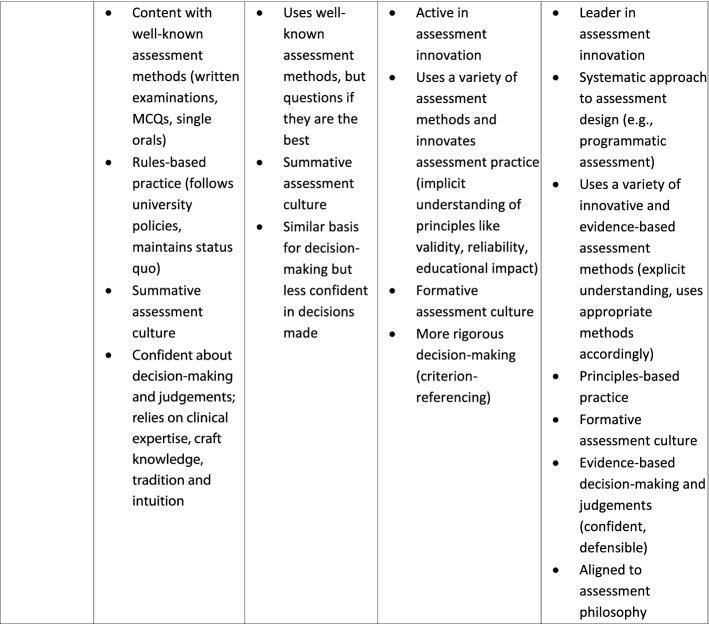
Clerkship conveners’ conceptions of assessment

Four categories were identified, representing collective, and distinctive yet hierarchically-inclusive variations in the experience and understanding of assessment. Each could be characterised across six dimensions. While not mutually exclusive, each dimension contributes something unique to the description of the conceptions.

The four conceptions were labelled: “Passive operator”, “Awakening inquirer”, “Active owner” and “Scholarly practitioner”. Six dimensions characterising the conceptions were: purpose of assessment, temporal perspective, role and responsibility, accountability and emotional valence (Table 4). Three other characteristics, broader than dimensions or conceptions, were identified that appeared to be strongly associated with, but may not have been intrinsic to describing, each conception: assessment literacy, professional identity, and self-efficacy.

### The “passive operator”

The “Passive operator” perceived assessment narrowly, as a concrete administrative task, and negatively, as an unavoidable burden. The primary purpose of assessment was summative, to obtain a mark for each student in a single course for the administrative purpose of passing a student with as little inconvenience, and the associated negative impact, as possible. This reflected a short-term (“today”) and local-scope understanding of assessment.

(1) “I see it somewhat unfortunately as a necessary stick in order to achieve a short-term goal of getting students to knuckle down and study during a brief exposure to us … How well students respond to the threat of a test … Assessment is necessary in terms of year mark, for the student to pass or whatever … The assessment of a particular year is purely for a short-term goal and has no real meaning for the student in terms of the following year.”

Passive operators relied on their clinical knowledge and experience in their assessment practice rather than assessment principles (quote 4). Accountability was expressed towards their profession, subsequently using assessment to measure disciplinary knowledge reproduction and gatekeep who may and may not enter their profession. A lack of reflexivity manifested as an uncritical acceptance and implementation of university assessment rules and standards, and maintenance of what has “always been done”. There was no sense of wanting to take ownership of or change assessment practice.

(2) “The university’s rules stipulate [it] … We go about it [assessment] the way we do purely because … the university recommends it.”

(3) “[The way we practice assessment is] probably historical … this is how we were examined and as a student that is what was expected of me and now in turn this is what I expect of my students.”

This conception was associated with a strong professional identity as clinician and low levels of assessment literacy and perceived self-efficacy. Clinical work was considered more important than educational roles and responsibilities.

(4) “I’m a clinician, I am not [an educator], I haven’t got any experience in education and my main interest is clinical work … most of the examiners are also just clinicians who are working in the various hospitals … so they are experienced but they are not trained … a lot of the examiners are very old-school in terms of having a mark that you can say this means a student is competent or not … Some examiners still don’t like tick sheets [rubric], so they’ll like listen to the story the student is telling them and then give a global impression without ticking.”

This conception manifested in practice as somewhat punitive, related to passive operators’ negative emotional experience with and view of assessment as an unwanted administrative requirement.

### The “awakening enquirer”

This conception extends the previous in viewing assessment not just as a summative tool for administrative purposes, but as one to measure student learning, albeit focused more on knowledge-reproduction than competency development.

(5) “Make sure that there is a certain standard of knowledge or certain level of knowledge.”

If feedback is given, it was merely quantitative as a grade, yet, there is a shift from the passive operator reflected as a change in perceived roles and responsibilities as an assessor, as seen in their accountability towards student learning.

In this conception, a realisation was evident that relying on implicit craft knowledge of assessment was no longer adequate. Awakening enquirers started questioning and critically reflecting on their assessment practice, recognising their limitations and revealing emerging reflexivity. A shift in emotional valence was observed; while assessment was seen as challenging, they were beginning to value it, expressed in a need for training in assessment to improve assessment literacy and self-efficacy.

(6) “[At a conference they said] ‘We are going to teach you how to do questions, how to standardise the process of the case and the questions.’ I already had some clinical scenarios and questions, but they made small changes that make a lot of sense right now. And one thing that I was extremely surprised by, the students enjoyed writing the test, because they told me, ‘Doctor before I would have to memorise to get the right answer, but now I have to think’ …. We are doctors, we are not teachers, nobody taught us how to teach and nobody taught us how to do a test … Somebody needs to teach us how to perform assessments in different ways.”

(7) “I think my knowledge in that regard is a bit too limited to tell you about the educational purpose (of assessment) … Then I realized [education is] actually a much-appreciated science, because I was actually kind of hesitant when I started it … I also realized is that I actually had very little idea of what to do … We actually had lectures about lecturers … that’s how I got involved, and I think because one starts to ask questions … For the first time [I] really looked beyond my beliefs and said, ‘That’s just how it works’ … I realized there is a theory behind this thing … The first time we started thinking about assessment … we learned about different assessment methods and so there I started thinking … It had value to me, but also frightened me a bit, in the sense that I knew I was lacking.”

Alongside the dimensions of the conceptions, there was a discernible shift in professional identity (quote 6), varying from a conflicted identity to some degree of balance in a dual clinician-educator identity.

(8) “I am a clinician convinced that the way I practice needs to have education in sight… Finding that halfway thing is something I believe.”

### The “active owner”

While sharing some dimensions with preceding conceptions, the “active owner” was informed and thoughtfully involved, displaying active ownership of their assessment from conceptualisation to practice. Active owners exhibited an overall more sophisticated understanding of assessment. Assessment was not conceptualised as being limited to a single course here and now, but shifted towards a longer-term (“tomorrow”) perspective, i.e., to facilitate a student’s longitudinal competency development, including their clinical skills and professional attitudes; additionally, as a summative tool for sound decision-making. Feedback was practiced with this in mind. Furthermore, the purpose of assessment extended from the psychometric to the moral and social aspects of assessment, exhibiting accountability towards ensuring safe patient care by future clinicians.

(9) “The requisite skills that are needed for them to be technically and diagnostically sufficient … We’re responsible to civil society. So, we train the students to assimilate their knowledge and technical skills that would make them good practitioners. Of course we hopefully give them other things such as empathy and ethical skills.”

(10) “[The medical school] does not prepare students to pass an exam, it’s for them to be good practitioners and to be competent … [The goal is] competence, competence, competence”.

Assessment was perceived positively, as important and valuable. Active owners displayed a sense of accountability towards students and their growth, which aligned with their perceived role of assessor and educator as a guide and support.

(11) “As a professor I can say, “Oh, it’s your fault, you’re not studying, it’s your fault” but it’s my responsibility we have to share … It’s a shared responsibility between students and teachers … we are in the same boat … We have to get feedback every single day, every single encounter, we have to give feedback … I have students come to my office … we will discuss, “Look, these are the great things you do and this is what we have to work on” … so that constant dialogue with a student is a feedback, it is not a grade, it is feedback.”

Greater reflexivity was inferred through active owners’ critical observations about and critiques of current assessment practices, and their desire for sound assessment practice. This was reflected in their desire to continue their professional development in assessment, for instance, through making time for self-study or attending formal training (see quote 6).

This conception was associated with a more advanced level of assessment literacy, supported through participants’ understanding and implementation of quality assessment principles and practices. Active owners understood that assessment served many purposes, requiring multiple and varied assessment methods to ensure more reliable and valid decision-making.

(12) “You should be assessed by multiple people … You evaluate every single thing, and I think it’s actually more … fair for the students … I believe in in multiple assessment and doing it a lot of times … I use a rubric.”

(13) “That mapping part I was talking [about] earlier, when the student knows which is the specific learning objectives for each learning opportunity ... So, the student starts reading the map … Those are the actions that are to be carried out by the student to achieve those particular learning objectives ... The student follows those things to get all the way to learn that learning objective.”

### The “scholarly practitioner”

The final conception is characterised by the expression of an overarching philosophy or paradigm of assessment. The principles of validity, reliability and educational impact were explicitly present in scholarly practitioners’ evidence-informed assessment understanding (e.g., programmatic assessment as a goal). Long-term and global perceptions saw assessment as a practice that should trigger lifelong learning, not just for the student, but for the educator too. Feedback was a dialogue, as much for the assessor as for the student (quote 11). Moreover, the outcomes of assessment in a course were expected to impact more broadly on the larger educational program, including teaching practices and curriculum design.

(14) “I think assessment is quality control of what we’re doing. So it goes both ways it goes, “What is the students doing wrong?” and “What we are doing wrong?” And we have tried to use the… all those assessments to see how we can improve our clerkship and what things we are doing wrong … Assessment goes both ways—with the professors and students.”

(15) “I’m going to give you [students] the tools that you are able to evolve in your learning of medical practice—and that’s done through a lifetime. If you stop learning you better quit.”

### Relationship with other conceptions of assessment

The second research question addressed the relationship between the conceptions of assessment identified here to those previously reported, the extent to which this framework accommodates previous findings. Numerous similarities were found (see Table 5). These included the range of purposes of assessment, along with temporal perspectives, from short-term administrative grade generation (Berendonk et al., 2013; Halinen et al., 2013), reproducing knowledge (Postareff et al., 2012; Samuelowicz & Bain, 2002) and summative (Brown, 2004) to the longer-term and formative transformation of understanding (Postareff et al., 2012; Samuelowicz & Bain, 2002) and development of learners (de Jonge et al, 2017; Halinen et al., 2013). In terms of alignment between roles and responsibilities, viewing oneself as a facilitator, to collaborate with students in their developmental journey, was seen (de Jonge et al., 2017; Halinen). The dimension of accountability towards institutions (Brown, 2004), external regulations (de Jonge et al., 2017), students (Berendonk et al., 2013; Brown, 2004), patients (de Jonge et al., 2017) and the educator, in terms of the quality of education more broadly (Brown, 2004), was shared. Degrees of reflexivity ranged from passively reproducing the ‘status quo’ (Halinen et al., 2013) to a critical awareness and engagement with assessment (Halinen et al., 2013), to reflective self-talk and action to transform assessment practice (Halinen et al., 2013; Postareff et al., 2012). Varied emotional valence attached to assessment were reported, from negative (Berendonk et al., 2013; Brown, 2004) to positive (Brown, 2004; Halinen et al., 2013).

**Table 5:** Alignment between literature, conceptions of assessment and associated factors

		Passive operator	Awakening inquirer	Active owner	Scholarly assessor
Purpose of assessment		Assessment as “irrelevant”—Brown (2004); Assessment serves no useful purpose; or assessment for administration (grading)—Berendonk et al. (2013); “Status quo”: assessment to obtain marks—Halinen et al. (2013); “Knowledge reproduction”: reproducing bits of knowledge and reproducing structured knowledge—Samuelowicz and Bain (2002)	Assessment for “student accountability” (summative assessment)—Brown (2004); “Awareness”: content- and learning-centred—Halinen et al. (2013); “Reproductive” conceptions of the purpose of assessment—Postareff et al. (2012); “Knowledge construction”: applying structured knowledge and organising subject knowledge—Samuelowicz and Bain (2002)	Assessment for “improvement” of student learning and teaching—Brown (2004); Assessment should stimulate learning—Berendonk et al. (2013); “Development”: (authentic) learning-centred, lifelong learning goal for all—Halinen et al., (2013, p.21); “Transformational” conceptions of the purpose of assessment—Postareff et al. (2012); “Knowledge transformation”: transforming disciplinary knowledge and transforming conceptions of discipline/world—Samuelowicz and Bain (2002)	
				“Agency”: assessment must guide self-regulated learning—de Jonge et al. (2017)	“Mutuality”: bi-directional/dialogic feedback between student and assessor—de Jonge et al. (2017)
Temporal Perspective		Short-term: obtain a mark—Berendonk et al. (2013) & Halinen et al. (2013)	–	Longer-term: learning -Berendonk et al., 2013	Long-term: accountability to patients and social service responsibility—de Jonge et al. (2017)
Role and Responsibility		–	–	–	Mutuality: collaborative relationship between student and teacher; co-learners—de Jonge et al. (2017) and Halinen et al. (2013)
Accountability		“Status quo”: institutional accountability and “School accountability”: quality control according to standards—Brown (2004); “Objectivity”: assessment needs to meet external regulations and objective standards—de Jonge et al. (2017)		“Development”: shared autonomy between students and teachers ("students as partners”) to achieve intended learning outcomes—Halinen et al. (2013)	
					“Accountability”: assessment needs to ensure high quality patient care—de Jonge et al. (2017)
			“Student accountability”: competency achievement for qualification and certification—Brown (2004) and Berendonk et al. (2013)		“Improvement”: assessments gives information about both students’ learning and educators’ teaching; how to improve education Brown (2004); “Adaptivity”: flexibility between external (objective competencies) and internal (clinical realities) regulations and standards—de Jonge et al. (2017)
Reflexivity		“Status quo”: reproduce the traditional way of doing things—Halinen et al. (2013)	“Awareness”: critical engagement (but no action)—Halinen et al. (2013)	“Development”: reflective self-talk, took action to change assessment practice—Halinen et al. (2013); “Transformational” conceptions as more reflective—Postareff et al. (2012)	
Emotional valence		“Assessment as irrelevant”—Brown (2004); negative (anxiety, doubt)—Berendonk et al. (2013)	–	“Conscious development of assessment”: motivated to transform awareness into action—Halinen et al. (2013); “Improvement”: assessment as necessary & valuable—Brown (2004)	
*Associated characteristics*					
Assessment literacy		Low: disciplinary content as important—Berendonk et al. (2013); Halinen et al. (2013) and Samuelowicz and Bain (2002)	Developing: assessment as a tool for knowledge construction—Samuelowicz and Bain (2002)	Sophisticated: assessment must be used for transforming disciplinary knowledge and transforming conceptions of discipline/world—Samuelowicz and Bain (2002); “Personal development”: expertise develops—Berendonk et al. (2013)	
Professional identity		“Status quo”: Teacher-centred—Halinen et al. (2013)	Dual teacher and assessor roles—Berendonk et al. (2013)	“Development”: educator/educationalist-centered—Halinen et al. (2013)	
Self-efficacy	“Assessor characteristics”: self-efficacy in own assessment abilities—Berendonk et al. (2013)				

There was also a notable coherence between the associated characteristics found here and those reported in literature. Varying levels of assessment literacy were reported: low to developing to sophisticated (Berendonk et al., 2013; Halinen et al., 2013; Samuelowicz & Bain, 2002). Differing professional identities were implied: teacher-centred (Halinen et al., 2013) which could relate to professional or disciplinary expert identity, a dual teacher-assessor identity (Berendonk et al., 2013) which could speak to the disciplinary-educator identity, and a more educator or educationalist-centred identity (Halinen et al., 2013) which could connect to a more scholarly assessor identity. Self-efficacy as an associated factor has not featured in other research on conceptions of assessment.

## Discussion

Four hierarchal conceptions, each elucidated by six descriptive dimensions, were progressively developed in exploring clerkship conveners’ conceptions of assessment in three diverse Southern settings. Although represented as categories for the purposes of description, these conceptions exist along a porous continuum from less to more complex. Conceptions of assessment were further associated with their assessment literacy, sense of professional identity, and perceived self-efficacy as factors moderating and/or moderated by, conceptions.

Conceptions of assessment have been explored at other times, in various contexts and different disciplines, without any attempt to craft a coherent view of these conceptions. Mapping previous conceptions against this work, revealed that our conceptions comprehensively encompass and expand previous models, for instance, through the addition of explicit and more nuanced descriptive dimensions of conceptions. These similarities are notable given the contextual differences between the settings, suggesting the potential for applicability across a range of contexts.

While alignment with previous work is extensive, the underlying origins of dimensions may differ between clinician-educators in Southern versus Northern settings. For instance, the administrative-focus of the “passive operator” could relate to the fact that clinician-educators in the global South are overwhelmed by the burden of disease and accompanying clinical workloads, leaving little room for educational thinking and activities, leading them to adopt a more operational frame of mind when it comes to assessment. On the other hand, an “active owner” who views the purpose of assessment for developing clinical competency, and feels a deep sense of accountability towards patients, could also be rooted in the realities of working in Southern settings where graduates are licensed for supervised clinical service—not necessarily because of an awareness of, or a desire to be aligned to, the latest medical education assessment scholarship.

Answering two questions would help evaluate the utility of these conceptions for supporting the professionalisation of assessment practice. Addressing the first, describing the relationship between assessment conceptions and practice, was not an explicit goal of this study but some data on this emerged as an artefact of the interview process (Table 2). These tentative observations, that there is a progression in assessment practice tracking with successive conceptions, are supported by other studies (Halinen et al., 2013; Norton, et al., 2019; Postareff et al., 2012; Watkins et al., 2005). If it is possible to support the progression of assessors’ conceptions along the continuum, this may then lead to improvements to assessment practice. Using faculty development to change educators’ conceptions of teaching has been found to be related to behaviour change that manifests as meaningful changes to teaching practice (Ho, 2000; Ho et al., 2001). Faculty development initiatives targeting conceptions may also lead to changed (improved) assessment practices (Offerdahl & Tomanek, 2011). Particularly in Southern and resource-constrained contexts (Table 2), developing the cohort of faculty who as individuals are largely responsible for high stakes assessments has particular import for quality improvement.

As with diagnostic assessment, understanding the conceptual starting points of individuals, before beginning faculty development, is useful as by knowing potential underlying assumptions, strengths to build on and gaps to address, faculty developers may be more effective in pitching their programmes at the correct level and in targeted and more personalised ways.

The second question, how conceptions emerge and develop, was also not explored here, yet a number of ‘associated characteristics’, that became apparent during the iterative interview process, appeared to track the progression through the hierarchical organisation of the outcome space. These characteristics suggest possible targets, likely among others, for intervention for conceptual and behavioural change. Assessment literacy, professional identity, and self-efficacy appeared to be influences that track conceptions. For instance, poor assessment literacy was associated with simplistic and negative conceptions of the purpose of assessment; whereas more advanced assessment literacy appeared to be associated with a more sophisticated conception of the purposes and temporal range of assessment and its outcomes. The notion has been advanced that increasing assessment literacy, for instance through faculty development, may aid movement along the continuum (Boud & Dawson, 2021). It is notable, though, that the relationship between literacy and practice may well be reciprocal (Offerdahl & Tomanek, 2011).

The potential role of professional identity is supported by findings about clinical teaching practice, with the development of stronger teacher identities being associated with improvements in teaching practice (Cantillon et al., 2016). A transition in professional identity was related to emerging reflexivity and involvement in educational scholarship (Adendorff, 2011)—which supports an argument for associated characteristics as antecedent factors in conceptions of assessment and potential targets for conceptual change.

Even if effective, attempts to change conceptions will not guarantee improved assessment practice. Disconnects between conceptions of assessment and assessment practice have been reported, due to the influence of additional factors (Bearman et al., 2016; Box et al., 2015; de Jonge et al., 2017; Offerdahl & Tomanek, 2011; Postareff et al., 2012). The relative impact of conceptions, and of other personal and contextual factors, and their interacting relationships, on assessment practice is being investigated.

Another priority for future research is the exploration of the applicability of these conceptions in other settings, and whether or not conceptions hold or fundamentally change based on context. As our conceptions enriched and deepened previous findings, so additional studies may extend our outcome space. For instance, would conceptions differ for assessors in rural settings? In Northern settings with more sophisticated assessment practices like programmatic assessment, do conceptions differ? Or do clinician educators as products of more recent times (in their educational experiences as students and/or academic faculty) in a larger practice of ‘assessment *for* learning’, possess more sophisticated conceptions of assessment?

The limitation of convenience sampling was countered through the sampling diversity of participants across different contexts. Moreover, while sampling was undertaken within “Southern” settings alone, seeking to address an epistemological and pragmatic gap in the literature, we acknowledge the problematic homogenisation of the terms “Southern” and the “global South”. The adoption of a “Southern” lens allowed us to critically reflect on previous work and position our study within the lived realities of global knowledge hierarchies. However, the alignment of our findings with previous work was somewhat surprising and brings the issues of delimiting participation according to context into question, and may undermine our questions around epistemological- and ecological-validity. Perhaps these findings confront our own misconceptions and prejudices, not just those of Northern researchers, of stereotyped “Southern” assessment perspectives and practices. However, intentionally-diverse sampling of clinician-educators, and educational practitioners more broadly, from both Southern and Northern settings could highlight potential epistemic and ecological differences in conceptions.

## Conclusion

Any intervention to change assessment practice to achieve desirable learning effects of assessment must involve the assessors. We propose that understanding assessors’ conceptions of assessment is an important part of the process needed to support changed assessment practice. The findings of this study extend our understanding of assessors’ conceptions in medical education and align with previously described conceptions of assessment from a variety of different educational levels, disciplines and contexts; offering a potentially productive avenue to explore professionalisation of assessment practice in various contexts.

### Supplementary Information

Below is the link to the electronic supplementary material.

Supplementary file1 (DOCX 36 KB)
