# Supplementary material for: Clinician educators’ conceptions of assessment in medical education
Source: Adv Health Sci Educ Theory Pract. 2023 Jan 20;28(4):1053–77. doi: 10.1007/s10459-022-10197-5 (PMC10624725; doi:10.1007/s10459-022-10197-5)
Supplement: Supplementary file 1 — Supplementary file1 (DOCX 36 KB) [file 10459_2022_10197_MOESM1_ESM.docx]

**Running head (shortened title)**: Conceptions of assessment

**Title**: Clinician educators’ conceptions of assessment in medical education

**Authors**: DA Sims^1^ FJ Cilliers^2^

**Affiliations**: ^1^University of the Western Cape, Cape Town, South Africa; ^2^Faculty of Health Sciences, University of Cape Town, Cape Town, South Africa

**Correspondence**: University of the Western Cape, 14 Blanckenberg Street, Bellville, South Africa; +27 021 959 2911; [dsims@uwc.ac.za](mailto:francois.cilliers@uct.ac.za)

**Appendix**: Interview guide exploring conceptions of assessment.

| **Opening questions:**   - What do you understand by the term “assessment”? How would you define “assessment”? - Describe your assessment practice: how do you practice assessment? - Why do you practice assessment? What is it for or what does it achieve?   **Examples of iterative prompts and follow-ups (evolved across interviews):**   - What do you believe is the purpose of your assessment practice? Summative assessment *of* learning, or formative assessment *for* learning? - What or who does your assessment impact on? (e.g., impact on student learning) What are the consequences of your assessment? (e.g., short-term, long-term) What is your assessment accountable towards? (e.g., patient, profession, institution, students) - How do you see yourself professionally? (e.g., clinician, educator, something else?) How do you see yourself as an assessor? What are your assessment responsibilities? - What assessment principles do you draw on to design your assessments and why? (e.g., validity, reliability, etc.) - What makes your assessment ‘sound’? How would you define a ‘good’ or ‘bad’ assessment? What are characteristics of a ‘good’ assessment and why? How do you determine the quality of an assessment? Do you evaluate your assessments – why/not? - What assessment methods do you use and why? Have you made any changes to your assessment practices? Why/not? - What do grades mean? How do you determine a ‘passing’ grade? Do you norm- or criterion-reference? Why? - What do you base your assessment decisions on? How confident do you feel about your assessment practice and why? - How do you feel about assessment? (e.g., negatively or positively) |
| --- |
